# Effect of Early Time-Restricted Eating on Metabolic Markers and Body Composition in Individuals with Overweight or Obesity

**DOI:** 10.3390/nu16142187

**Published:** 2024-07-09

**Authors:** Dalila Rubí Mena-Hernández, Guadalupe Jiménez-Domínguez, José D. Méndez, Viridiana Olvera-Hernández, Mirian C. Martínez-López, Crystell G. Guzmán-Priego, Zeniff Reyes-López, Meztli Ramos-García, Isela E. Juárez-Rojop, Selene S. Zavaleta-Toledo, Jorge L. Ble-Castillo

**Affiliations:** 1 Centro de Investigación, División Académica de Ciencias de la Salud (DACS), Universidad Juárez Autónoma de Tabasco (UJAT), Villahermosa 86150, Mexico; 2 Departamento de Medicina Interna, Hospital General de Zona No. 46, Instituto Mexicano del Seguro Social (IMSS), Villahermosa 86060, Mexico; 3 Hospital de Cardiología, Centro Médico Nacional Siglo XXI, Instituto Mexicano del Seguro Social (IMSS), Ciudad de México 06703, Mexico

**Keywords:** early time-restricted eating, overweight, obesity, metabolic markers, body composition

## Abstract

This study aimed to evaluate the effect of early time-restricted eating (eTRE) on metabolic markers and body composition in individuals with overweight or obesity. Seventeen subjects completed a randomized, crossover, and controlled clinical trial. Twelve women and five men participated, with a mean age of 25.8 ± 10.0 years and a BMI of 32.0 ± 6.3 kg/m^2^. The eTRE intervention included 16 h of fasting (3:00 pm to 7:00 am) and 8 h of ad libitum eating (7:00 am to 03:00 pm) (16:8). The trial included four weeks of interventions followed by a four-week washout period. Body weight, waist and hip circumferences, and body composition measurements were taken. Additionally, a venous blood sample was collected for biochemical determinations. In a before–after analysis, eTRE induced a reduction in BW and BMI in women but this was not significant when compared to the control group. eTRE did not modify any other anthropometric measurements, fasting biochemical parameters, glycemic and insulinemic responses, blood pressure, or subjective appetite. In conclusion, eTRE did not induce beneficial effects on the glycemic and lipid metabolisms, body composition, subjective appetite, or blood pressure. These findings may be attributed to the special characteristics of the population and the short intervention period.

## 1. Introduction

Obesity is a complex and multifactorial disease whose major contributor is nutritional imbalance [1,2]. Its prevalence has been increasing over the years, leading to it being considered an epidemic and a public health problem [3]. Obesity contributes directly to incident cardiovascular risk factors such as dyslipidemia, hypertension, type 2 diabetes, and sleep disorders. This critical situation has led to expanded dietary interventions to limit energy intake and promote weight loss [4]. Lifestyle interventions are the first line of treatment to combat obesity and metabolic diseases [5], and they involve quality and quantitative dietary modifications and increased physical activity.

Diet interventions to prevent obesity are classified into two types: calorie restriction, which involves a 25% reduction in the daily caloric intake and usual timing of mealtimes; and intermittent fasting (IF), where the eating period is restricted [6]. IF is a diet term for three different types of diets: alternate day fasting, the 5:2 diet, and time-restricted eating (TRE) [7]. Alternate day fasting (ADF) involves a fasting day (energy severely restricted) and an alternated “feast day” (ad libitum intake). The 5:2 diet includes five days per week of eating normally and two fasting days that might be consecutive or non-consecutive. Finally, TRE is a regimen that differs from the above, where individuals fast for 12 to 20 h per day, and their ad libitum eating window is reduced to 4 to 12 h [8,9].

Early time-restricted eating (eTRE) is a strategy where food is restricted to the first hours in the morning to afternoon and can align the central and peripheral circadian clock, causing changes in the regulation of the metabolism [6,10]. After a fasting period of 8 to 12 h, the liver begins to break down fatty acids to ketone bodies as a fuel source to maintain vital organs and tissues, which may improve metabolic markers, glucose regulation, and body weight [11,12].

A recent study evaluated the effects of eTRE on the metabolism of men with prediabetes using an 18 h fasting period and 6 h eating window, resulting in improved insulin sensitivity, blood pressure, oxidative stress, and appetite [13]. Similarly, other studies in healthy subjects showed a reduction in the 24 h mean blood glucose, body weight, adiposity, and inflammation in subjects under 08:00–14:00 and 06:00–15:00 eating windows for 5 weeks and 4 days, respectively [14,15]. In contrast, other studies have found minimal effects of TRE on metabolic markers and body composition. In particular, a 16 h fasting period for three months showed no significant changes in blood biomarkers associated with metabolic and cardiovascular risk in middle-aged women with obesity [16]. Likewise, other authors reported that midday eating (12:00–20:00) did not alter metabolic markers and body weight in patients with overweight and obesity [17]. Therefore, the evidence of the effects of eTRE on metabolic markers is still controversial. The present study aims to evaluate the effect of eTRE on metabolic markers and body composition in individuals with overweight and obesity.

## 2. Materials and Methods

### 2.1. Ethics Statement

This study was approved by the institutional research ethics committee of the Juárez Autonomous University of Tabasco (CIEI-1184). Likewise, the clinical trial was registered on the Australian New Zealand Clinical Trials Registry (ACTRN;12623001311640) and conducted according to the Helsinki Declaration. All participants who agreed to participate in the study were asked to sign an informed consent form for their inclusion.

### 2.2. Participants

Participants were recruited in Villahermosa Tabasco, Mexico, through posters and social media from September to December 2023. Potential participants were invited to an evaluation session where the characteristics and objectives of the study were explained in detail. Subsequently, they were selected based on their medical history, blood pressure, and anthropometric measurements. In addition, a blood sample was taken to confirm the eligibility criteria. The inclusion criteria were as follows: (1) men and women between 18 and 50 years old; (2) BMI > 25 kg/m^2^; (3) stable body weight for three months before the study (gain or loss <4 kg); (4) physical activity (<150 min per week); (5) a regular menstrual cycle (women); and (6) the ability to independently provide informed consent. The exclusion criteria were as follows: (1) a diagnosis of diabetes, cardiovascular disease, or kidney, liver, or gastrointestinal disease; (2) being pregnant or breastfeeding; (3) use of drugs that might affect metabolism (antidiabetic medications, steroids, thyroid hormones, beta-blockers, adrenergic stimulating agents); (4) fasting >15 h/day; (5) nicotine/tobacco habitual consumption in the preceding three months; (6) alcohol consumption more than twice a week; (7) engaged in regular sports or rigorous physical activity.

### 2.3. Study Design and Protocol

We conducted a randomized, controlled, crossover clinical trial. Before starting, participants were randomized to participate in two dietary interventions: control (ad libitum food intake without restriction in eating schedule) or eTRE (ad libitum eating during no more than 8 h between 07:00 and 15:00 and fasting for the rest of the day) for 4 weeks and culminating with the alternative intervention group after a 4-week washout period. eTRE participants were encouraged to drink plenty of water and were also allowed to consume energy-free beverages.

Before and after each intervention phase, anthropometric measurements were taken from patients, including body weight, waist and hip circumference, waist-to-hip ratio (WHR), body composition, and a venous blood sample for biochemical determinations. We also measured their blood pressure and performed a meal tolerance test (MTT) with the evaluation of subjective appetite using the Flint Visual Analog Scale (VAS) [18]. Before MTT, subjects were instructed to fast for at least 12 h and to refrain from strenuous exercise, alcohol, caffeine, and tobacco for at least 24 h.

### 2.4. Adherence

To improve compliance, a daily reminder of the eating window’s end time point was sent to participants, along with a form where participants uploaded photos of their meals and drinks consumed. In addition, weekly phone calls were made for monitoring and following up. A form to record adverse events and attachment difficulties during the eTRE phase was also sent.

We considered good adherence when participants had more than 85% compliance with the assigned eTRE intervention days. Participants with less than this percentage were excluded from the data analysis.

### 2.5. Body Composition and Blood Pressure

The body composition of patients was obtained by bioelectrical impedance (Tanita, TBF-300A, Mexico City, Mexico). A portable stadiometer measured their height, and a fine metric was used to measure the waist (upper edge of the iliac crest) and hip circumference (gluteal prominence). BMI (weight (kg)/height^2^ (m)) and waist-hip ratio (WHR, waist circumference/hip circumference, in cm) values were calculated from these measurements. Heart rate, systolic, and diastolic blood pressure were measured using a digital sphygmomanometer (Omron, HEM-7120, Kyoto, Japan), with readings taken individually for each arm and then averaged.

### 2.6. Meal Tolerance Test

A meal tolerance test was performed one day before the beginning and one day after each intervention. A catheter was inserted into the antecubital vein in the participants’ forearm for blood sample collection. Immediately after the catheter placement, the first blood sample was taken (t = −5) and another was taken 5 min later (t = 0). Subsequently, participants were offered a standardized breakfast consisting of supplementary food (Simisure^®^; Modern strategies; Mexico City, Mexico) (296 mL, 120 kcal; 6 g protein, 1 g lipids, and 21.5 g carbohydrates) and a Kellogg’s^®^ Rice Krispies^®^ bar (Kellogg’s Company, Battle Creek, MI, USA), which had to be consumed at least within 5 min. A 22 g bar of Kellogg’s^®^ Rice Krispies^®^ contains a total of 90 kcal, protein <1 g, total fat 2 g, and total carbohydrates 17 g. The supplementary food and the cereal bar added up to a total of 210 kcal, 7 g of protein, 38.5 g of carbohydrates, and 3 g of lipids. Additional blood samples were drawn at 15, 30, 60, 90, and 120 min after consuming the standardized breakfast to determine blood glucose and insulin concentrations.

### 2.7. Appetite Assessment

To assess subjective appetite sensation, a validated VAS was used [18]. These were 100 mm in length and anchored with words at each end expressing the most positive and most negative ratings. Hunger, satiety, fullness, and prospective food consumption were assessed. Questions were asked as follows: (1) How hungry do you feel? (2) How satisfied do you feel? (3) How full do you feel? (4) How much food do you think you could eat? Hunger is defined as a feeling that drives the desire to eat or indicates the need for food. Satiety is the perception of not having an immediate need for food intake between meals, which refers to the state of inhibition of eating, and fullness is the sensation of the degree of heaviness of the stomach that leads to stopping eating. The VAS was applied 5 minutes before obtaining the MTT blood samples, which were collected at 0, 15, 30, 60, 90, and 120 min in relation to breakfast consumption.

### 2.8. Biochemical Measurements

Fasting blood samples from the antecubital vein were collected and centrifuged, and the serum was separated for the determination of glucose, cholesterol, HDL-C, triglycerides, and insulin concentrations. Other blood samples were collected during the MTT time points specified above to determine glucose and insulin. In all cases, samples that were not immediately analyzed were stored at −70 °C until further analysis.

Glucose, cholesterol, HDL-C, and triglycerides determinations were performed using an enzymatic–colorimetric assay in a clinical biochemistry autoanalyzer (Spinreact; Spin-200E, Girona, Spain) following the Spinreact Laboratory instructions. Insulin was measured by immunoassay of chemiluminescent microparticles using Wiener Lab equipment (Wiener Lab, CLIA-900, Rosario, Argentine) following the instructions provided by the manufacturer. Insulin resistance (IR) at fasting was estimated by the index homeostatic model assessment (HOMA-IR), calculated as the product of fasting glucose (mg/dL) and insulin (μU/mL), divided by 405 [19]. LDL-C concentrations were calculated using the formula of Friedewald: LDL-C = C total-HDL-C − triglycerides/5 [20].

### 2.9. Statistical Analysis

The primary hypothesis was that eTRE would result in a lower glycemic response compared to the control group. We estimated a sample size of 16 participants using PASS 21.0 software (Kaysville, UT, USA) to detect a difference of 10% in the primary outcome variable of the area under the curve (AUC) for glucose to obtain a power of 95% and a type I error of 5% in a crossover design. Thse AUCs for both glucose and insulin were calculated as the 120 min AUC value, divided by the 120 min duration of the MTT. Randomization was carried out by generating random numbers at https://www.random.org/ (accessed on 8 January 2024). Data were expressed as mean ± standard deviation or unless otherwise specified. The D’Agostino and Pearson normality test was performed to assess whether the data were consistent with a Gaussian distribution. Two-way analysis of variance (ANOVA) in combination with Tukey’s post hoc test was used to determine differences in fasting biochemical markers. Time-course data were analyzed by repeated measures (RMs) two-way ANOVA to assess the effects of treatment, time, and the interaction of treatment and time. A Tukey post hoc test was used for comparisons. Statistical analyses were conducted with the statistical software GraphPad Prism (GraphPad Software, San Diego, CA, USA) version 9.0 for Windows. *p*  <  0.05 was adopted as a significant difference.

## 3. Results

### 3.1. Participants

**Figure 1:**
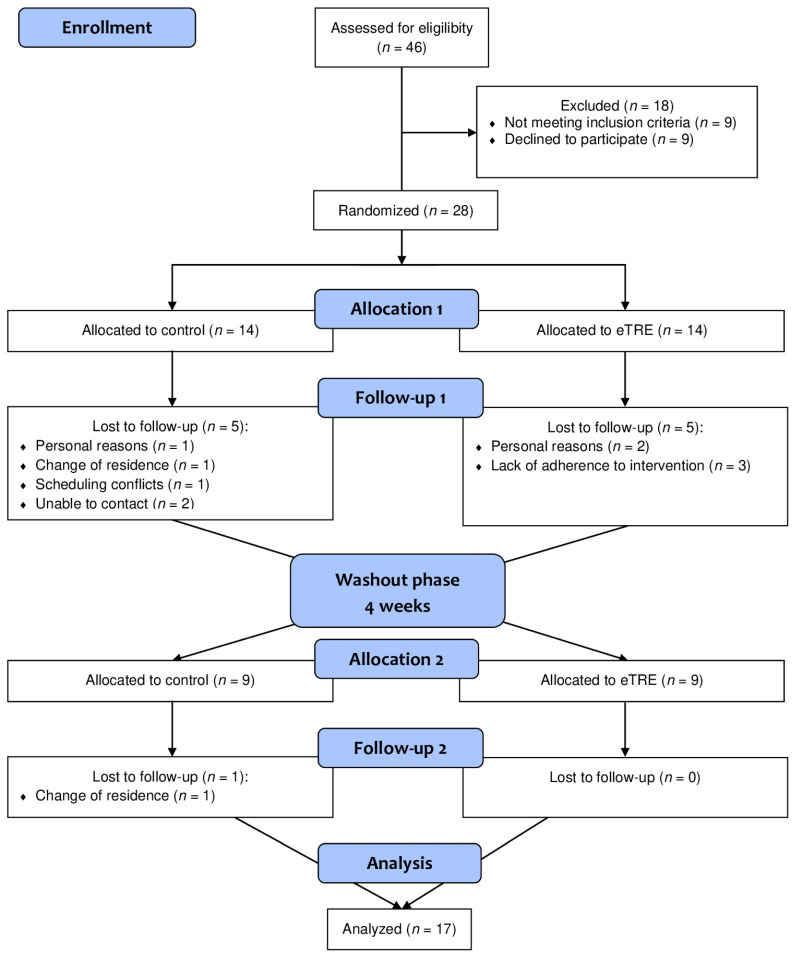
CONSORT diagram showing the participant flow through the study.

Figure 1 shows the flow of participants throughout the study. A total of 46 volunteers were recruited for the study. Of these, 28 participants were selected and randomly assigned to receive one of the two interventions. Eight participants withdrew for different reasons, three due to personal reasons, two due to changes in residence, two due to a loss of contact, and one due to conflicts with their work schedule. A total of 20 participants completed both intervention periods; however, three of them were eliminated from the final analysis due to a lack of adherence. Finally, 17 participants (70.6% women and 29.4% men) with a mean age of 25.7 ± 10.0 (SD) years and a BMI of 32.0 ± 6.3 kg/m^2^ were included in the analysis. Out of these, 11 were aged 18–24 years, which means that they are considered young adults.

Table 1 presents the clinical and anthropometric characteristics of the subjects who completed the study. Nine (53%, eight women and one man) had obesity with BMI > 30 kg/m^2^; eight had overweight (47%, four women and four men), with BMI values between 25 and 29.9 kg/m^2^. Among the subjects with obesity, nine had high waist circumference measurements (eight women >88 cm and one man >102 cm). Five patients (three men and two women) had increased systolic and diastolic blood pressure levels (>130 mmHg and >85 mmHg, respectively). On the other hand, altered fasting glucose was found only in one subject (>100 mg/dL). Finally, four patients (two women and two men) had increased triglyceride levels (>150 mg/dL), and eleven had decreased HDL-C levels (ten women and one man, with values <50 mg/dL and <40 mg/dL, respectively).

### 3.2. Effect of eTRE on Fasting Biochemical Parameters

eTRE did not induce changes in the fasting concentrations of the different biochemical markers (Table 2) of glucose (*p* = 0.75), insulin (*p* = 0.53), total cholesterol (*p* = 0.92), triglycerides (*p* = 0.93), HDL-C (*p* = 0.98), LDL-C (*p* = 0.89), or HOMA-IR (*p* = 0.46).

### 3.3. Effect of eTRE on Body Composition and Blood Pressure

No significant changes were found in body weight, BMI, fat percentage, fat mass, lean mass, waist and hip circumference, or WHR after the eTRE intervention when compared to the control group. However, a reduction in body weight (BW) and BMI was observed in women after the eTRE intervention in a before–after statistical analysis (*p* = 0.006 and *p =* 0.009, respectively) (Supplementary Table S1). Moreover, the levels of systolic and diastolic blood pressure, as well as heart rate, were not modified (Table 2).

### 3.4. Effect of eTRE on Glycemic Response

**Figure 2:**
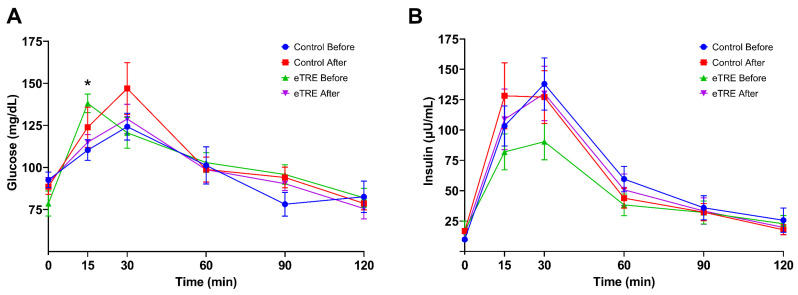
Postprandial response of glucose (**A**) and insulin (**B**) during MTT. Data are expressed as mean ± standard error of the mean. Comparisons based on two-way repeated measures ANOVA. * *p* = 0.02; *n* = 10.

During the MTT, the glycemic and insulin responses were evaluated, and no effects of eTRE were observed when compared to the control group (*p* > 0.05, RM two-way ANOVA) (Figure 2). However, a reduction in glucose levels was observed at the 15 min time point when comparing before and after the intervention (*p* = 0.02, RM two-way ANOVA and Tukey’s test). It should be noted that the sample size (*n*) in this analysis was reduced to 10 participants due to difficulties in taking blood samples.

### 3.5. Effect of eTRE on Subjective Appetite

**Figure 3:**
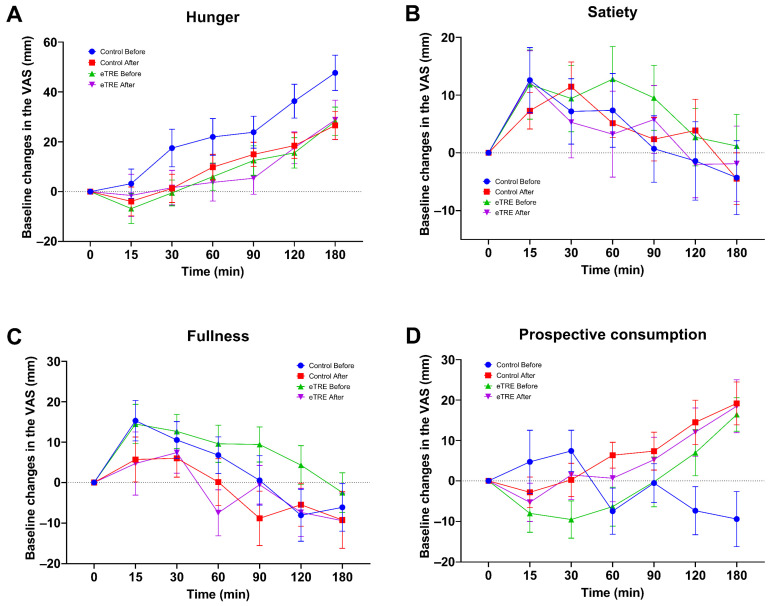
Evaluation of the subjective sensation of hunger (**A**), satiety (**B**), fullness (**C**), and prospective consumption (**D**). Data are expressed as mean ± standard error of the mean. Comparisons based on two-way repeated measures ANOVA. *n* = 17.

After the ANOVA analysis, no changes were found in the subjective sensations of hunger *p* = 0.14, satiety *p* = 0.91, fullness *p* = 0.40, or prospective consumption *p* = 0.30 (Figure 3). 

## 4. Discussion

In recent years, eTRE has become more popular for preventing and treating metabolic disorders. Indeed, there are many studies demonstrating its efficacy in improving anthropometric and cardiometabolic health markers in individuals with excessive weight and obesity-related metabolic disturbances [3,6,10,21,22,23,24]. However, in the present study, no beneficial effects of eTRE on the glycemic and lipid metabolisms, body composition, subjective appetite, and blood pressure were observed.

Regarding the effects of eTRE on the glycemic metabolism, a beneficial effect was expected, since it is recognized that during the fasting period, macro- and micronutrients are less accessible to cells and thus decrease adiposity and insulin resistance through reduced caloric intake and metabolic reprogramming. The reduction in insulin production and increased AMPK levels have been proposed as a mechanism to improve insulin sensitivity and glucose homeostasis [25,26]. The improvement in fasting glycemia has been consistently observed in several clinical studies and meta-analyses [3,6,10,15,24,27,28]. Likewise, another meta-analysis found beneficial effects on fasting insulin [24].

In this study, however, no effects of eTRE on fasting glycemia and insulin were observed, and consequently, no effects on the HOMA-IR index were found either. Nevertheless, other authors such as Cienfuegos et al. found a reduction in insulin resistance using 18 and 20 h of fasting [29]. Similarly, Xie et al. reported a decrease in the HOMA-IR index after 16 h of fasting with an eating window between 06:00 and 15:00 [15].

Our results also showed no effects of eTRE on the glycemic and insulin responses, which is consistent with some reports [30,31,32]; however, they contrast with other studies, such as some with crossover designs and intervention periods as short as one week (two-week washout) or six weeks (two-week washout), which found significant reductions in the glycemic response [33,34]. Likewise, in another crossover study that used five weeks of intervention with a seven-week washout period, a decrease in the insulin response was found [13]. Furthermore, there are few studies evaluating the glycemic and insulin responses using an MTT instead of an oral glucose tolerance test (OGTT) [33]. Here, we used MTT, considering that a standardized mixed meal containing protein and lipids in addition to carbohydrates would further induce a greater physiological stimulus on insulin secretion because of their effects on pancreatic β cells [35].

The 16:8 eTRE used in this study did not affect the biomarkers of lipid metabolism. Although the effects on lipids are still controversial, the main eTRE effect has been observed in the reduction in fasting triglycerides [6,24,30,33,36]. Furthermore, several meta-analyses have consistently found no changes in the fasting total cholesterol, LDL-C, and HDL-C levels [3,6,10,24]. The absence of effects in our study could be partially associated with the fact that the serum lipid levels in most of our participants were within normal reference values under baseline conditions.

Of note, eTRE induced a moderate but significant reduction in BW and BMI values in women during the before–after statistical analysis (*p* = 0.006 and *p* = 0.009, respectively). This group lost 1.0 Kg BW and their BMI value reduced by 1.0 after four weeks of the intervention period. This result is important, since it is known that even a small weight reduction can lower the risk of heart disease, inflammation, hypertension, and type 2 diabetes [37]. Moreover, this finding serves as evidence of good subject compliance during the eTRE intervention. Despite this finding, the positive effect of eTRE was not maintained when the eTRE effect was compared with the control group in a two-way RM ANOVA. A possible explanation for this might be that these participants acquired healthier dietary habits during the experiment, which prevented them from regaining weight during the ad libitum eating period. The beneficial effects of eTRE on body composition and anthropometric measurements have been consistently reported by other groups [6,10,24]. However, others have failed to find these effects even after 5, 8, and 12 weeks of eTRE intervention [13,17,28]. These controversial findings may be attributed to diverse reasons such as heterogeneity among subjects and variations in the implemented interventions.

The subjective appetite measurements did not show significant changes. It remains unclear how eTRE influences subjective appetite, as few studies have assessed it and the results are contradictory [13,31,34,38]. Unlike our approach, these studies evaluated the subjective sensation of appetite at different times of the day during the intervention periods. The effect of eTRE on appetite hormone levels also remains unclear [13,15,33,38]. We consider that the null effect found in our study could be associated with the possibility that the implemented eating window and/or intervention period were insufficient to achieve synchrony between the peripheral clock and the central clock, thereby failing to reset the secretion of appetite hormones.

Likewise, systolic and diastolic blood pressure and heart rate levels were not modified after eTRE. To date, it is unclear whether this intervention produces changes in blood pressure, because only a few studies have reported reductions in systolic blood pressure levels. Still, to the best of our knowledge, none of them have shown a significant effect on the diastolic blood pressure or heart rate [6,13,39].

The absence of a statistically significant overall effect of eTRE on various variables studied in the present investigation may be partially attributed to the early-stage metabolic alterations of the participants. The population we studied included 11 young adults under 24 years of age. Typically, in young individuals with a short disease evolution period, metabolic disturbances such as fasting hyperglycemia or glucose intolerance have not yet appeared. It is known that these pathologies may take up to 15 years to develop after the onset of insulin resistance [40]. In our study, only one subject had altered fasting glycemia; none of them presented glucose intolerance, defined as 2 h postprandial glycemia between 140 and 199 mg/dL, which is considered a prediabetes status [41,42]; and only 35% of them (six subjects) met other criteria for metabolic syndrome according to the ATP III classification [43]. Thus, most of the participants could be classified as having “metabolically healthy obesity”.

Few studies have been performed in young adults aged 18 to 24, with the majority focusing on subjects above 30 years of age [3,6,10,24,36,44,45,46,47]. Obesity among this age group, referred to as “emerging adulthood”, is a growing concern due to its increasing prevalence and the unique challenges that it poses [48,49]. This age group is marked by significant lifestyle changes and the acquisition of dietary habits that can contribute to a permanent trend of weight gain and lead to far-reaching consequences on individual health and well-being and societal costs [48,50].

Although the subjects’ compliance in this study was supported by the BW reduction observed in the female group under eTRE, it is crucial for future research to maintain strict control over the participants’ caloric intake. In this study, however, the informed data on food records was not reliable because the incomplete descriptions. It is known that self-reports of dietary intake sometimes differ or underestimate the true energy intake value, thus potentially providing misleading analyses [51,52]. The reduction in caloric intake has been considered a principal factor and previous studies have reported that typically, eTRE decreases caloric intake by at least ∼300 kcal/d [29,39,53]. In this study, we do not rule out the possibility that our participants had excessive caloric consumption during the eTRE intervention. Another possible reason for the reduced eTRE e effects in our study could be the short duration of the intervention, as most of the observed positive effects have been observed in longer periods of at least twice as much time as ours [28,29,36].

This study has several limitations, including our small sample size, the short duration of the intervention period, the use of bioelectrical impedance analysis to estimate body composition, which is less accurate than other methods, the lack of estimation of caloric intake, and the inability to measure serum ketone body concentration and appetite hormones, which could have helped confirm adherence in the eTRE group.

Despite these limitations, our study also possesses several strengths. Firstly, we included a group of young adult subjects, a population that has been insufficiently studied in the context of eTRE interventions. Secondly, our study design was crossover and randomized, which reduces the influence of confounding covariates, since each participant serves as their own control. Thirdly, we evaluated glycemic and insulin responses through a mixed-meal tolerance test (MTT), which can induce a greater incretin response compared to other testing methods. Lastly, to the best of our knowledge, this is the first study to evaluate the effect of eTRE in the Mexican population.

The effect of eTRE remains controversial, underscoring the importance of conducting further studies to clarify its impact and elucidate the optimal schedule and duration required to observe its effects. Additionally, we recommend that future studies focus on increasing the sample size, extending the intervention period, and evaluating additional factors such as appetite hormone levels and serum ketone bodies.

## 5. Conclusions

Four weeks of 16:8 eTRE did not induce changes in glycemic and lipidic markers, body composition, subjective appetite, and blood pressure levels in adults with overweight and obesity. These effects could be attributed to the special characteristics of the population and the short intervention period.

## Figures and Tables

**Table 1:** Characteristics of the participants at baseline.

Variable	All Participants (*n* = 17)	Women (*n* = 12)	Men (*n* = 5)
Age (years)	25.7 ± 10.0	20.0 (18.3–33.3)	21.0 (19.5–38.0)
Body weight (kg)	85. 4 ± 19.6	85.3 ± 22.8	83.2 (73.9–94.4)
BMI (kg/m^2^)	32.0 ± 6.3	33.0 ± 7.1	29.1 (27.1–31.9)
BMI 25–29.9 kg/m^2^	8 (47%)	4 (33%)	4 (80%)
BMI >30 kg/m^2^	9 (53%)	8 (67%)	1 (20%)
% Body fat	36.7 ± 9.6	38.8 ± 9.1	27.6 (21.9–35.1)
Fat mass (kg)	26.50 (20.4–37.3)	33.9 ± 16.2	24.0 (17.0–32.7)
Lean mass (kg)	54.2 ± 10.7	47.1 (43.4–57.5)	63.0 (56.8–63.7)
Waist circumference (cm)	92.9 ± 12.9	91.8 ± 14.0	99.0 (85.3–104.0)
SBP (mmHg)	120.3 ± 15.5	117.3 ± 16.7	131.5 (118.5–135.0)
DBP (mmHg)	78.0 ± 10.3	77.3 ± 10.2	83 (67.8–89.5)
Fasting glucose (mg/dL)	82.0 ± 10.8	81.4 ± 8.8	83.5 (68.1–98.9)
Fasting insulin (μU/mL)	4.6 (3.7–6.5)	4.4 (3.6–5.6)	6.2 (4.5–8.9)
Total cholesterol (mg/dL)	171.5 ± 30.7	174.9 ± 31.3	155.0 (137.5–195.2)
Triglycerides (mg/dL)	97.1 (68.5–152.5)	106.9 ± 50.6	110.0 (72.4–236.8)
HDL-C (mg/dL)	42.2 ± 6.2	43.9 ± 5.0	33.8 (32.7–47.0)
HOMA-IR	2.1 (1.7–4.3)	2.0 (1.6–3.6)	3.3 (1.5–7.2)

Values are expressed as mean ± SD; median (percentile 25–percentile 75); and *n* (%). Abbreviations: BMI: body mass index, SBP: systolic blood pressure, DBP: diastolic blood pressure, HDL-C: high-density lipoprotein cholesterol, HOMA-IR: homeostasis model assessment—insulin resistance.

**Table 2:** Effect of eTRE on metabolic markers, body composition, and physiological parameters.

Variable	*n*	eTRE		Control		*p* Value
		Before	After	Before	After	
Metabolic markers						
Glucose (mg/dL)	15	84.7 ± 8.8	89.7 ± 9.1	89.4 ± 14.3	87.4 ± 12.9	0.75
Insulin (μU/mL)	15	8.2 (4.0–16.6)	14.4 (12.1–18.2)	11.8 ± 8.6	18.8 ± 8.0	0.53
Total cholesterol (mg/dL)	15	162.1 (144.0–203.8)	188.4 (140.5–198.9)	174.6 ± 37.6	170.5 ± 34.9	0.92
Triglycerides (mg/dL)	15	103.0 (71.0–144.5)	125.1 (62.0–186.8)	98.4 (63.6–155.1)	104.3 (79.0–136.0)	0.93
HDL-C (mg/dL)	15	44.7 ± 8.0	41.5 ± 8.2	42.5 ± 6.6	43.6 ± 11.4	0.98
LDL-C (mg/dL)	15	102.6 ± 23.5	107.3 ± 31.0	106.7 ± 30.6	100.6 ± 30.5	0.89
HOMA-IR	15	1.2 (0.7–2.9)	3.2 (2.6–4.3)	2.0 (1.3–3.6)	3.9 (3.0–5.8)	0.46
Body composition						
Body weight (kg)	17	85.3 ± 19.6	84.7 ± 19.5	85.0 ± 19.5	84.9 ± 19.3	0.99
BMI (kg/m2)	17	31.9 ± 6.4	31.7 ± 6.3	31.8 ± 6.3	31.8 ± 6.2	0.98
% Body fat	17	36.1 ± 9.6	36.7 ± 9.6	37.2 ± 9.4	36.9 ± 9.4	0.84
Fat mass (kg)	17	26.5 (20.8–37.3)	28.5 (21.0–39.4)	32.7 ± 15.2	32.5 ± 15.2	0.78
Lean mass (kg)	17	50.0 (43.7–58.5)	54.8 (44.1–59.0)	52.3 ± 8.4	50.7 ± 9.5	0.84
Waist circumference (cm)	17	90.0 (82.0–97.0)	92.0 (80.0–101.5)	91.3 ± 13.7	92.1 ± 12.8	0.82
Hip circumference (cm)	17	114.2 ± 16.3	115.1 ± 13.7	114.1 ± 13.9	114.9 ± 14.8	0.98
WHR	17	0.8 ± 0.1	0.8 ± 0.1	0.8 ± 0.1	0.8 ± 0.1	0.71
Physiological parameters						
SBP (mmHg)	17	116.0 (107.5–123.0)	115.0 (111.0–119.5)	118.4 ± 14.4	113.1 ± 13.1	0.84
DBP (mmHg)	17	77.5 ± 10.5	76.9 ± 8.2	78.8 ± 10.3	75.0 ± 8.6	0.92
Heart rate (bpm)	17	77.7 ± 12.3	75.7 ± 11.0	75.7 ± 14.5	76.9 ± 12.8	0.90

Values are expressed as mean ± SD and median (percentile 25–percentile 75). Abbreviations: HDL-C: high-density lipoprotein cholesterol, LDL-C: low-density lipoprotein cholesterol, HOMA-IR: homeostasis model assessment—insulin resistance, BMI: body mass index, WHR: waist–hip ratio, SBP: systolic blood pressure, DBP: diastolic blood pressure. *p* values represent the result of comparing the effect of eTRE to the control using a two-way repeated measures ANOVA.

## Data Availability

The dataset used in this publication is available from the corresponding author on reasonable request. Data are not available publicly due to privacy reasons.

## Supplementary Materials

The following supporting information can be downloaded at: https://www.mdpi.com/article/10.3390/nu16142187/s1, Table S1: Effect of eTRE on metabolic markers, body composition, and physiological parameters.

## References

[1] Chooi Y.C., Ding C., Magkos F. (2019). The epidemiology of obesity. Metabolism.

[2] Obesity and Overweight. https://www.who.int/news-room/fact-sheets/detail/obesity-and-overweight.

[3] Pureza I., Macena M.L., da Silva Junior A.E., Praxedes D.R.S., Vasconcelos L.G.L., Bueno N.B. (2021). Effect of early time-restricted feeding on the metabolic profile of adults with excess weight: A systematic review with meta-analysis. Clin. Nutr..

[4] Kahan S. (2016). Overweight and obesity management strategies. Am. J. Manag. Care.

[5] Ryan D.H., Kahan S. (2018). Guideline recommendations for obesity management. Med. Clin. N. Am..

[6] Moon S., Kang J., Kim S.H., Chung H.S., Kim Y.J., Yu J.M., Cho S.T., Oh C.M., Kim T. (2020). Beneficial effects of time-restricted eating on metabolic diseases: A systemic review and meta-analysis. Nutrients.

[7] Welton S., Minty R., O’Driscoll T., Willms H., Poirier D., Madden S., Kelly L. (2020). Intermittent fasting and weight loss: Systematic review. Can. Fam. Physician.

[8] Elortegui Pascual P., Rolands M.R., Eldridge A.L., Kassis A., Mainardi F., Lê K.A., Karagounis L.G., Gut P., Varady K.A. (2023). A meta-analysis comparing the effectiveness of alternate day fasting, the 5:2 diet, and time-restricted eating for weight loss. Obesity.

[9] Patterson R.E., Sears D.D. (2017). Metabolic effects of intermittent fasting. Annu. Rev. Nutr..

[10] Pellegrini M., Cioffi I., Evangelista A., Ponzo V., Goitre I., Ciccone G., Ghigo E., Bo S. (2020). Effects of time-restricted feeding on body weight and metabolism. A systematic review and meta-analysis. Rev. Endocr. Metab. Disord..

[11] Anton S.D., Moehl K., Donahoo W.T., Marosi K., Lee S.A., Mainous A.G., Leeuwenburgh C., Mattson M.P. (2018). Flipping the metabolic switch: Understanding and applying the health benefits of fasting. Obesity.

[12] Li Z., Heber D. (2021). Intermittent fasting. JAMA.

[13] Sutton E.F., Beyl R., Early K.S., Cefalu W.T., Ravussin E., Peterson C.M. (2018). Early time-restricted feeding improves insulin sensitivity, blood pressure, and oxidative stress even without weight loss in men with prediabetes. Cell Metab..

[14] Jamshed H., Beyl R.A., Della Manna D.L., Yang E.S., Ravussin E., Peterson C.M. (2019). Early time-restricted feeding improves 24-hour glucose levels and affects markers of the circadian clock, aging, and autophagy in humans. Nutrients.

[15] Xie Z., Sun Y., Ye Y., Hu D., Zhang H., He Z., Zhao H., Yang H., Mao Y. (2022). Randomized controlled trial for time-restricted eating in healthy volunteers without obesity. Nat. Commun..

[16] Schroder J.D., Falqueto H., Mânica A., Zanini D., de Oliveira T., de Sá C.A., Cardoso A.M., Manfredi L.H. (2021). Effects of time-restricted feeding in weight loss, metabolic syndrome and cardiovascular risk in obese women. J. Transl. Med..

[17] Lowe D.A., Wu N., Rohdin-Bibby L., Moore A.H., Kelly N., Liu Y.E., Philip E., Vittinghoff E., Heymsfield S.B., Olgin J.E. (2020). Effects of time-restricted eating on weight loss and other metabolic parameters in women and men with overweight and obesity: The treat randomized clinical trial. JAMA Intern. Med..

[18] Flint A., Raben A., Blundell J.E., Astrup A. (2000). Reproducibility, power and validity of visual analogue scales in assessment of appetite sensations in single test meal studies. Int. J. Obes. Relat. Metab. Disord..

[19] Matthews D.R., Hosker J.P., Rudenski A.S., Naylor B.A., Treacher D.F., Turner R.C. (1985). Homeostasis model assessment: Insulin resistance and beta-cell function from fasting plasma glucose and insulin concentrations in man. Diabetologia.

[20] Friedewald W.T., Levy R.I., Fredrickson D.S. (1972). Estimation of the concentration of low-density lipoprotein cholesterol in plasma, without use of the preparative ultracentrifuge. Clin. Chem..

[21] Cornelissen G. (2012). When you eat matters: 60 years of Franz Halberg’s Nutrition Chronomics. Open Nutraceuticals J..

[22] Marcheva B., Ramsey K.M., Buhr E.D., Kobayashi Y., Su H., Ko C.H., Ivanova G., Omura C., Mo S., Vitaterna M.H. (2010). Disruption of the clock components clock and BMAL1 leads to hypoinsulinaemia and diabetes. Nature.

[23] Singh R.B., Cornelissen G., Mojto V., Fatima G., Wichansawakun S., Singh M., Kartikey K., Sharma J.P., Torshin V.I., Chibisov S. (2020). Effects of circadian restricted feeding on parameters of metabolic syndrome among healthy subjects. Chronobiol. Int..

[24] Kamarul Zaman M., Teng N., Kasim S.S., Juliana N., Alshawsh M.A. (2023). Effects of time-restricted eating with different eating duration on anthropometrics and cardiometabolic health: A systematic review and meta-analysis. World J. Cardiol..

[25] Cienfuegos S., McStay M., Gabel K., Varady K.A. (2022). Time restricted eating for the prevention of type 2 diabetes. J. Physiol..

[26] Vasim I., Majeed C.N., DeBoer M.D. (2022). Intermittent fasting and metabolic health. Nutrients.

[27] Chair S.Y., Cai H., Cao X., Qin Y., Cheng H.Y., Ng M.T. (2022). Intermittent fasting in weight loss and cardiometabolic risk reduction: A randomized controlled trial. J. Nurs. Res..

[28] Peeke P.M., Greenway F.L., Billes S.K., Zhang D., Fujioka K. (2021). Effect of time restricted eating on body weight and fasting glucose in participants with obesity: Results of a randomized, controlled, virtual clinical trial. Nutr. Diabetes.

[29] Cienfuegos S., Gabel K., Kalam F., Ezpeleta M., Wiseman E., Pavlou V., Lin S., Oliveira M.L., Varady K.A. (2020). Effects of 4- and 6-h time-restricted feeding on weight and cardiometabolic health: A randomized controlled trial in adults with obesity. Cell Metab..

[30] Chow L.S., Manoogian E.N.C., Alvear A., Fleischer J.G., Thor H., Dietsche K., Wang Q., Hodges J.S., Esch N., Malaeb S. (2020). Time-restricted eating effects on body composition and metabolic measures in humans who are overweight: A feasibility study. Obesity.

[31] Haganes K.L., Silva C.P., Eyjólfsdóttir S.K., Steen S., Grindberg M., Lydersen S., Hawley J.A., Moholdt T. (2022). Time-restricted eating and exercise training improve HBA1C and body composition in women with overweight/obesity: A randomized controlled trial. Cell Metab..

[32] Liu D., Huang Y., Huang C., Yang S., Wei X., Zhang P., Guo D., Lin J., Xu B., Li C. (2022). Calorie restriction with or without time-restricted eating in weight loss. N. Engl. J. Med..

[33] Hutchison A.T., Regmi P., Manoogian E.N.C., Fleischer J.G., Wittert G.A., Panda S., Heilbronn L.K. (2019). Time-restricted feeding improves glucose tolerance in men at risk for type 2 diabetes: A randomized crossover trial. Obesity.

[34] Martens C.R., Rossman M.J., Mazzo M.R., Jankowski L.R., Nagy E.E., Denman B.A., Richey J.J., Johnson S.A., Ziemba B.P., Wang Y. (2020). Short-term time-restricted feeding is safe and feasible in non-obese healthy midlife and older adults. Geroscience.

[35] Lages M., Barros R., Moreira P., Guarino M.P. (2022). Metabolic effects of an oral glucose tolerance test compared to the mixed meal tolerance tests: A narrative review. Nutrients.

[36] Moro T., Tinsley G., Bianco A., Marcolin G., Pacelli Q.F., Battaglia G., Palma A., Gentil P., Neri M., Paoli A. (2016). Effects of eight weeks of time-restricted feeding (16/8) on basal metabolism, maximal strength, body composition, inflammation, and cardiovascular risk factors in resistance-trained males. J. Transl. Med..

[37] ElSayed N.A., Aleppo G., Aroda V.R., Bannuru R.R., Brown F.M., Bruemmer D., Collins B.S., Hilliard M.E., Isaacs D., Johnson E.L. (2022). 8. Obesity and weight management for the prevention and treatment of type 2 diabetes: Standards of care in diabetes—2023. Diabetes Care.

[38] Ravussin E., Beyl R.A., Poggiogalle E., Hsia D.S., Peterson C.M. (2019). Early time-restricted feeding reduces appetite and increases fat oxidation but does not affect energy expenditure in humans. Obesity.

[39] Gabel K., Hoddy K.K., Haggerty N., Song J., Kroeger C.M., Trepanowski J.F., Panda S., Varady K.A. (2018). Effects of 8-hour time restricted feeding on body weight and metabolic disease risk factors in obese adults: A pilot study. Nutr. Healthy Aging.

[40] Phillips L.S., Ratner R.E., Buse J.B., Kahn S.E. (2014). We can change the natural history of type 2 diabetes. Diabetes Care.

[41] American Diabetes Association Professional Practice Committee (2022). 2. Classification and diagnosis of diabetes: Standards of medical care in diabetes—2022. Diabetes Care.

[42] De Sanctis V., Daar S., Soliman A.T., Tzoulis P., Karimi M., Di Maio S., Kattamis C. (2022). Screening for glucose dysregulation in β-thalassemia major (β-tm): An update of current evidences and personal experience. Acta Biomed..

[43] Grundy S.M., Brewer H.B., Cleeman J.I., Smith S.C., Lenfant C. (2004). Definition of metabolic syndrome: Report of the National Heart, Lung, and Blood Institute/American Heart Association conference on scientific issues related to definition. Circulation.

[44] Isenmann E., Dissemond J., Geisler S. (2021). The effects of a macronutrient-based diet and time-restricted feeding (16:8) on body composition in physically active individuals-a 14-week randomised controlled trial. Nutrients.

[45] Jones R., Pabla P., Mallinson J., Nixon A., Taylor T., Bennett A., Tsintzas K. (2020). Two weeks of early time-restricted feeding (etrf) improves skeletal muscle insulin and anabolic sensitivity in healthy men. Am. J. Clin. Nutr..

[46] Naguib M.N., Hegedus E., Raymond J.K., Goran M.I., Salvy S.J., Wee C.P., Durazo-Arvizu R., Moss L., Vidmar A.P. (2022). Continuous glucose monitoring in adolescents with obesity: Monitoring of glucose profiles, glycemic excursions, and adherence to time restricted eating programs. Front. Endocrinol..

[47] Vidmar A.P., Naguib M., Raymond J.K., Salvy S.J., Hegedus E., Wee C.P., Goran M.I. (2021). Time-limited eating and continuous glucose monitoring in adolescents with obesity: A pilot study. Nutrients.

[48] Ellison-Barnes A., Johnson S., Gudzune K. (2021). Trends in obesity prevalence among adults aged 18 through 25 years, 1976–2018. JAMA.

[49] Katsoulis M., Lai A.G., Diaz-Ordaz K., Gomes M., Pasea L., Banerjee A., Denaxas S., Tsilidis K., Lagiou P., Misirli G. (2021). Identifying adults at high-risk for change in weight and bmi in england: A longitudinal, large-scale, population-based cohort study using electronic health records. Lancet Diabetes Endocrinol..

[50] Poobalan A., Aucott L. (2016). Obesity among young adults in developing countries: A systematic overview. Curr. Obes. Rep..

[51] Dhurandhar N.V., Schoeller D., Brown A.W., Heymsfield S.B., Thomas D., Sørensen T.I., Speakman J.R., Jeansonne M., Allison D.B. (2015). Energy balance measurement: When something is not better than nothing. Int. J. Obes..

[52] Schoeller D.A., Thomas D., Archer E., Heymsfield S.B., Blair S.N., Goran M.I., Hill J.O., Atkinson R.L., Corkey B.E., Foreyt J. (2013). Self-report-based estimates of energy intake offer an inadequate basis for scientific conclusions. Am. J. Clin. Nutr..

[53] Gill S., Panda S. (2015). A smartphone app reveals erratic diurnal eating patterns in humans that can be modulated for health benefits. Cell Metab..

