# Supplementary material for: Effect of Early Time-Restricted Eating on Metabolic Markers and Body Composition in Individuals with Overweight or Obesity
Source: Nutrients. 2024 Jul 9;16(14):2187. doi: 10.3390/nu16142187 (PMC11279456; doi:10.3390/nu16142187)
Supplement: Supplementary file 1 [file nutrients-16-02187-s001.zip › nutrients-3089350-supplementary.pdf]

Supplementary Table S1. Effect of eTRE on metabolic markers, body composition, and physiological parameters.

Variable	eTRE						Control						*p** value
	Women		*p* value	Men		*p* value	Women		*p* value	Men		*p* value	
	Before	After		Before	After		Before	After		Before	After		
Metabolic markers													
Glucose (mg/dL)	86.4 ± 7.8	89.4 ± 9.6	0.43	80.0 (72.6–90.4)	87.0 (82.9–99.5)	0.31	90.8 ± 15.22	87.6 ± 9.5	0.43	83.5 (74.6–100.0)	86.3 (69.0–104.9)	0.88	0.75
Insulin (μU/mL)	11.7 ± 9.5	15.6 ± 7.5	0.07	8.2 (4.8–13.4)	13.2 (10.1–27.5)	0.31	10.3 ± 5.8	19.0 6.4	0.005	7.6 (4.9–28.6)	12.8 (9.2–30.7)	0.06	0.53
Total cholesterol (mg/dL)	176.0 ± 30.6	175.2 ± 38.7	0.90	155.0 (141.8–183.2)	194.9 (141.9–207.7)	0.81	176.0 ± 41.2	167.4 ± 34.0	0.42	186.1 (137.5–199.2)	194.4 (139.0–206.0)	0.44	0.92
Triglycerides (mg/dL)	96.6 (78.5–149.4)	100.9 (56.4–133.6)	0.72	110.0 (48.5–229.8)	186.8 (109.5–290.0)	0.06	104.7 ± 48.8	98.2 ± 25.9	0.63	115.0 (72.4–245.6)	149.0 (82.7–340.3)	0.06	0.93
HDL-C (mg/dL)	45.0 ± 6.9	42.5 ± 6.9	0.21	48.7 (32.7–53.5)	38.7 (28.8–50.9)	0.13	43.5 ± 6.0	44.1 ± 6.6	0.82	44.1 (31.8–47.0)	32.5 (28.1–62.3)	>0.99	0.98
LDL-C (mg/dL)	108.8 ± 23.0	111.1 ± 31.1	0.72	99.9 (68.6–106.8)	95.1 (69.1–132.8)	0.81	111.6 ± 34.0	103.7 ± 27.7	0.41	93.8 (76.0–130.8)	104.5 (60.1–123.7)	0.63	0.89
HOMA-IR	0.9 (0.7 – 3.6)	3.3 (2.6–4.6)	0.08	1.6 (1.1–2.5)	2.9 (2.2–6.2)	0.19	2.4 (1.2–3.6)	3.9 (3.1–4.6)	0.07	1.6 (1.1–6.1)	3.5 (1.6–6.7)	0.13	0.46
Body composition													
Body weight (kg)	85.6 ± 22.7	84.6 ± 22.5	0.006	83.2 (76.2–93.9)	83.3 (75.6–95.4)	>0.99	84.8 ± 22.8	84.8 ± 22.6	0.98	85.0 (77.1–93.5)	85.5 (77.1–92.7)	0.69	0.99
BMI (kg/m^2)	33.1 ± 7.1	32.7 ± 7.1	0.009	29.1 (26.7–31.7)	29.2 (26.5–32.2)	>0.99	32.8 ± 7.1	32.8 ± 7.1	0.87	29.8 (27.1–31.6)	29.6 (27.0–31.5)	0.63	0.98
% Body fat	39.0 ± 9.3	40.1 ± 8.3	0.63	29.1 (22.8–35.1)	28.8 (20.8–36.1)	0.69	41.0 ± 7.5	40.2 ± 7.5	0.30	27.6 (21.9–34.4)	29.4 (20.2–37.2)	0.63	0.84

Fat mass (kg)	34.3 ± 16.6	35.8 ± 16.2	0.49	25.0 (17.4–32.7)	25.8 (15.7–33.4)	>0.99	36.2 ± 16.2	35.6 ± 16.2	0.35	24.0 (17.0–32.2)	25.4 (15.6–34.3)	0.81	0.78
Lean mass (kg)	47.4 (43.4–57.1)	45.7 (42.5–55.5)	0.07	57.1 (42.8–62.5)	61.8 (56.4–63.3)	0.50	48.6 ± 7.3	49.2 ± 6.7	0.21	60.5 (59.1–63.2)	61.1 (42.0–63.8)	0.44	0.84
Waist circumference (cm)	92.0 (80.3–95.0)	92.5 (79.5–100.8)	0.76	88.0 (81.5–103.5)	91.0 (83.0–104.0)	0.25	89.2 ± 14.1	91.6 ± 13.1	0.13	97.0 (85.3–107.5)	89.0 (81.5–107.5)	0.69	0.82
Hip circumference (cm)	116.1 ± 18.9	117.3 ± 15.2	0.54	108.0 (103.5–117.0)	113.0 (101.0–117.0)	>0.99	116.7 ± 15.4	117.2 ± 16.7	0.51	104.0 (102.0–116.0)	108.0 (103.5–116.5)	0.38	0.98
WHR	0.8 ± 0.08	0.8 ± 0.06	0.52	0.8 (0.8–0.9)	0.9 (0.8–0.9)	0.38	0.8 ± 0.06	0. 08 ± 0.05	0.24	0.9 (0.8–1.0)	0.8 (0.8–1.0)	0.19	0.71
Physiological parameters													
SBP (mmHg)	112.0 (106.3–119.8)	114.0 (102.0–116.8)	0.36	125.0 (114.0–136.0)	121.0 (114.5–133.0)	0.56	114.4 ± 14.0	111.3 ± 11.7	0.38	132.0 (116.5–137.5)	118.0 (102.5–131.5)	0.19	0.84
DBP (mmHg)	76.8 ± 10.0	77.0 ± 8.4	0.95	83.0 (66.0–90.0)	79.0 (68.5–84.0)	0.88	79.2 ± 10.7	75.8 ± 6.5	0.21	79.0 (68.0–88.0)	77.0 (60.0–84.0)	0.25	0.92
Heart rate (bpm)	77.7 ± 11.1	77.3 ± 10.5	0.86	77.0 (62.5–94.0)	67.0 (62.0–85.8)	0.13	74.0 (70.3–83.8)	79.0 (71.8–86.3)	0.66	75.0 (53.5–86.0)	68.0 (58.5–88.5)	0.56	0.90

Values are expressed as mean ± SD; median (percentile 25–percentile 75). Abbreviations: HDL-C: high-density lipoprotein cholesterol, LDL-C: low-density lipoprotein cholesterol, HOMA-IR: homeostasis model assessment-insulin resistance, BMI: body mass index, WHR: waist-hip ratio, SBP: systolic blood pressure, DBP: diastolic blood pressure. p values represent the result of comparing the values before-after interventions using paired Student's test or Wilcoxon. p* values represent the result of comparing the effect of eTRE to the control using a two-way repeated measures ANOVA. For metabolic markers: women n = 10, men n = 5; for body weight composition and physiological parameters: women n = 12, men n = 5.
